# Adrenocortical Gap Junctions and Their Functions

**DOI:** 10.3389/fendo.2016.00082

**Published:** 2016-06-29

**Authors:** Cheryl L. Bell, Sandra A. Murray

**Affiliations:** ^1^Department of Cell Biology, University of Pittsburgh School of Medicine, Pittsburgh, PA, USA

**Keywords:** ACTH, connexin, gap junction plaques, gap junction vesicles, steroidogenesis

## Abstract

Adrenal cortical steroidogenesis and proliferation are thought to be modulated by gap junction-mediated direct cell–cell communication of regulatory molecules between cells. Such communication is regulated by the number of gap junction channels between contacting cells, the rate at which information flows between these channels, and the rate of channel turnover. Knowledge of the factors regulating gap junction-mediated communication and the turnover process are critical to an understanding of adrenal cortical cell functions, including development, hormonal response to adrenocorticotropin, and neoplastic dedifferentiation. Here, we review what is known about gap junctions in the adrenal gland, with particular attention to their role in adrenocortical cell steroidogenesis and proliferation. Information and insight gained from electrophysiological, molecular biological, and imaging (immunocytochemical, freeze fracture, transmission electron microscopic, and live cell) techniques will be provided.

## Introduction

The adrenal is a complex gland that is histologically and functionally two tissues, the cortex and medulla, within a connective tissue capsule (1). Not only the two regions of the adrenal gland are from different embryonic origins but also the cells of the medulla are composed of cells that have a neuroendocrine function, while the cells of the cortex are epithelial cells that function in endocrine metabolism. There is evidence that cells of both the cortex and the medulla are regulated by cell–cell communication of regulatory molecules through membrane channels, called gap junctions (2–8). The efficiency of the adrenal gland, as well as other endocrine glands, to respond to stimulation is thought to depend not only on hormone receptor interaction but also on intercellular communication through gap junctions.

Gap junctions in the adrenal gland, as in other tissues, provide low-resistance pathways for the direct intercellular exchange of small molecules (9, 10). In early years, gap junctions and cell communication were mainly studied with electron microscopic (11–13), electrophysiological (14), and fluorescent dye transfer (15, 16) techniques. More recently, however, the proteins (connexins) composing the gap junction pore have been identified, and the tissue distribution of the different connexin family members has been demonstrated (17–20). Further, the molecular details of the assembly of connexins into functional gap junction channels, the involvement of kinases in this assembly, and the architectural arrangement of connexins into functional pore complexes have been described (21, 22).

In this review, we present a historical summary of gap junctions in the adrenal cortex from their discovery with imaging and electrophysiological techniques to current studies of the connexin types, distribution, abundance, and turnover. The role of gap junctions in the adrenal cortical response to adrenocorticotropin (ACTH) will be discussed, and the fundamental concepts and implication of gap junctions in steroidogenesis, proliferation, and cancer will be analyzed. Finally, gap junction-mediated cross talk between the cells of the adrenal cortex and medulla will be discussed, as it relates to adrenocortical function. We will begin with a brief review of gap junctions in the adrenal cortex.

## Characterization of Gap Junctions in the Adrenal Cortex

**Figure 1:**
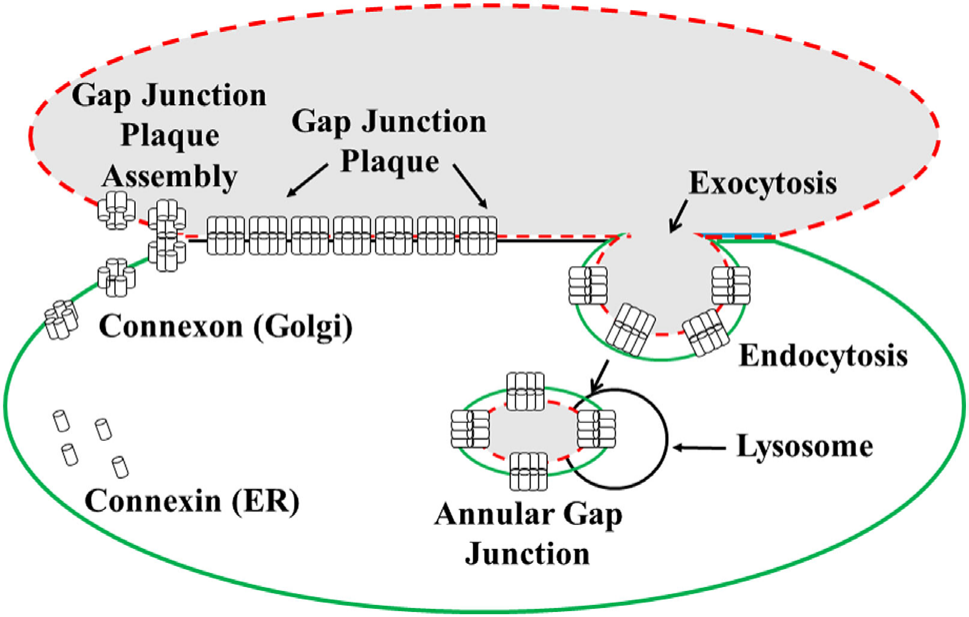
**Illustration of the formation and degradation of gap junction plaques and annular gap junctions**. Connexin proteins synthesized in the endoplasmic reticulum (ER) oligomerize to form connexon complexes. The connexons are transported to the cell surface and inserted into the plasma membrane where they form hemichannels. These hemichannels can dock with hemichannels of an apposing cell and cluster to form a gap junction plaque, characterized by a 2–4 nm gap between the two cell membranes. Gap junction plaques are removed from the cell surface through endoexocytosis, which results in the formation of an annular gap junction. The annular gap junction is then degraded through lysosomal proteolysis [modified from Ref. (130)].

**Figure 2:**
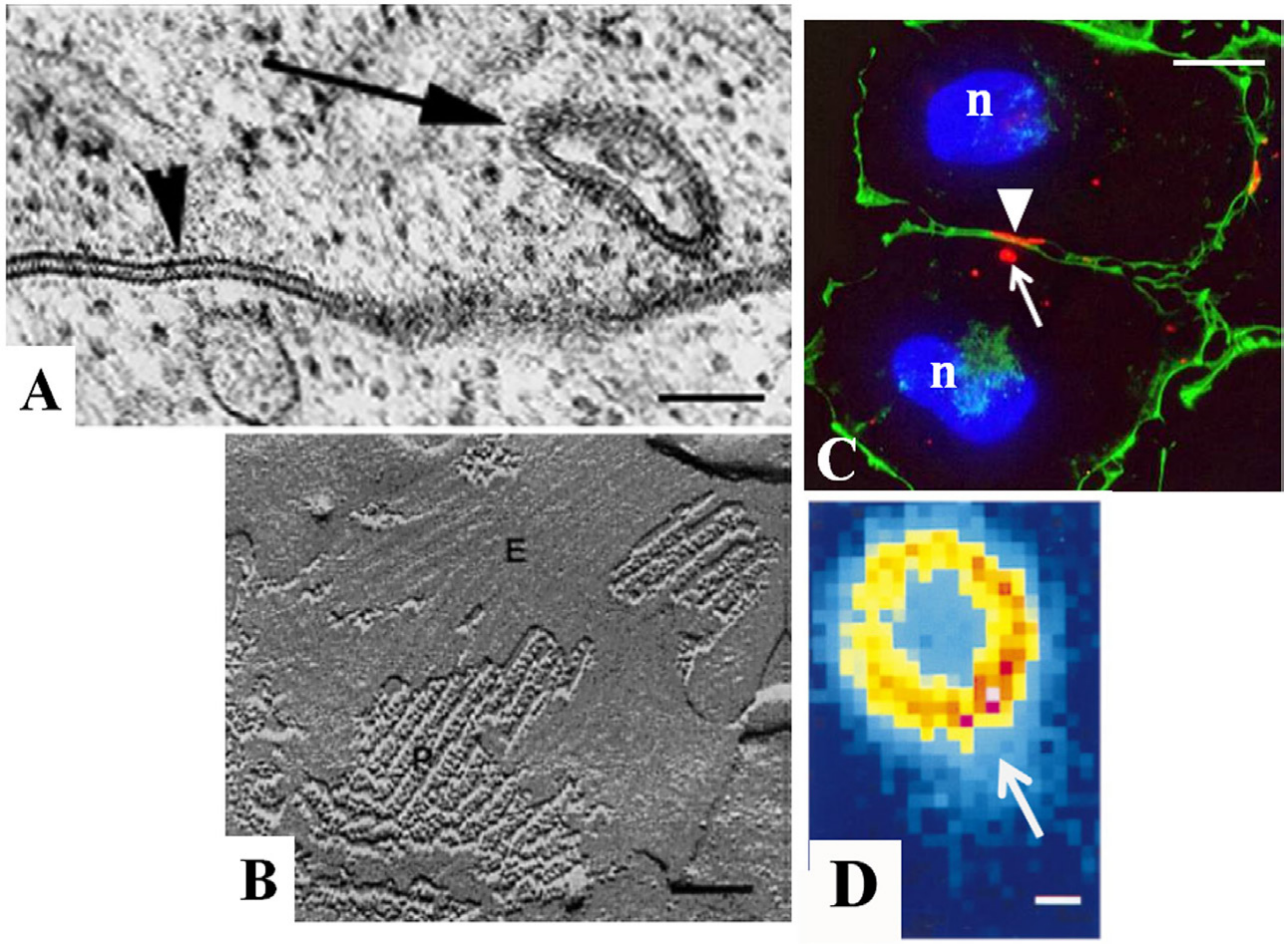
**Characterization of gap junctions in SW-13 adrenocortical tumor cells**. The size, location, and structure of gap junction plaques (arrowheads) and annular gap junctions (arrows) have been determined with **(A)** transmission electron microscopy, **(B)** freeze-fracture electron microscopy, **(C)** immunofluorescence, and **(D)** confocal microscopy. The protoplasmic (P) and extracellular (E) fracture faces are shown in the freeze-fracture replica of the gap junction plaque in **(B)**. Cell borders are defined by cortical actin (green) in **(C)**. Note the lumen of the annular gap junction revealed with confocal microscopy in **(D)**. n, nucleus. Bars: **(A)** 100 nm, **(B)** 60 nm, **(C)** 10 μm, and **(D)** 0.3 μm. [**(A)** from Ref. (48), **(B)** from Ref. (130), **(C)** from Ref. (131), and **(D)** from Ref. (132)].

Gap junctions occur between the membranes of two closely opposed cells and are characterized by the pairing of intramembranous connexin complexes across a 2–4 nm gap (Figures 1 and 2) (23). The first transmission electron microscopic images of gap junction plaques in the adrenal gland were acquired from fetal rat adrenals in 1970 (24) and more extensively from adult glands of a number of animals in 1972 (11, 25). Gap junctions in the adrenal resembled those of other tissues and were positively identified by the presence of the characteristic pentalaminar membrane and the 2–4 nm gap separating the membrane of two adjacent cells (Figure 2A). The area of membrane covered by gap junction plaques (plaque size) and the packing pattern of adrenal cortical cell gap junction channels could be obtained with freeze-fracture electron microscopy (11). With this technique, the adrenal cell membrane bilayer was split in the hydrophobic plane (26), and the typical protoplasmic (P)-fracture face and extracellular (E)-fracture face distribution of membrane particles was used to identify gap junctions (Figure 2B). Gap junctions could be distinguished by the observed clustering of 8.5 nm particles on the P-fracture face and pits on the E-fracture face (27, 28) (Figure 2B). With freeze-fracture techniques, the size of gap junction plaques was demonstrated in the rat adrenal cortex to be larger and more abundant than those found in most other tissues of the body, especially those plaques in the area of the adrenal cortex near or juxtaposed to the medulla (24). Gap junction plaques have now been described in the adrenal from a vast number of different mammalian species (6, 29–31). An important advantage of freeze-fracture imaging was the capacity to confirm that the adrenal cortical gap junctions were composed of aggregates of intramembranous particles that were paired with one another across the intercellular space. The observation that these paired particles extended across the gap, seen with transmission electron microscopy, provided structural evidence for the existence of channels, which could serve as means for movement of molecules between cells. However, the critical role of gap junctions as channels for communication was established by experiments in which it was demonstrated that hepatocytes and myocardial cells only passed current if gap junctions were observed and if gap junctions were not present, current did not pass between cells (32).

The movement of current was first demonstrated in the adrenal cortex with electrophysiological techniques in experiments, which used adult and fetal rabbit adrenal gland slices (10). Although such studies confirmed that material was capable of communicating between cells in the adrenal, the function of this movement was not addressed in these early studies. In addition, information on the possible size of molecules that could move between the gap junction channels was provided from dye transfer studies in which molecules smaller than 1,000 Da could communicate between cells while larger molecules were excluded (16, 33, 34). Based mainly on the early electrophysiological and dye transfer studies, as well as transmission electron microscopic and freeze-fracture images, it was hypothesized that gap junctions allowed the passage of molecules between adjacent cells, and thus, modulate adrenal cortical cell population growth and hormonal response (8, 35). However, the molecules composing and regulating the gap junction pore and, more important, the definitive role of gap junction-mediated communication in adrenal cortical function remained to be defined.

Our knowledge concerning the role of gap junctions, in general, was greatly enhanced by the isolation and characterization of the connexin proteins that composed gap junction pores. It has become clear that the particles seen with freeze-fracture electron microscopy are the gap junction channels and that each gap junction channel consists of 12 connexin molecules, 6 from one cell docked to 6 in the adjacent cell. The clustering of these channels forms the gap junction plaque (27, 36).

The production of antibodies directed at the connexin proteins allowed the detection of gap junction protein types with immunofluorescence and western blot techniques (19, 20, 37). In humans, there are 21 different connexin types that differ by their amino acid sequences and molecular weights (38). Connexin 43 (Cx43) gap junction protein was demonstrated as the major, if not only, connexin gap junction protein type in the adrenal cortex (5, 30, 31, 39, 40). While investigators have also reported Cx26, Cx32, and Cx50 in humans (41), their expression has not been reported in other mammals (3, 6, 7, 42). It should be noted, however, that of the 21 known connexin family members (38), only 6 have been extensively evaluated in the adrenal cortex, and it is possible that others will be detected with further analysis.

With immunofluorescence microscopy, the distribution of Cx43 could be quickly detected and its distribution more reliably compared with adrenal zones than with conventional transmission electron microscopy (5, 30, 40, 43). The Cx43 gap junctions in the adrenal cortex appear as small puncta or longer plaques on the cell surface between contacting cells (Figures 2C,D). The cells of the adrenal cortex are polyhedral in shape, and their three-dimensional relationship to one another has been revealed with serial section transmission electron microscopy (44) and scanning electron microscopy of freeze-cracked adrenal glands (45). Based on the three-dimensional imaging provided by these techniques, gap junctions are thought to form at smooth “contact” sites and on facets on microvilli of cells (45). The facets from two cells were observed to be in close apposition, and it is here as well as at the smooth sites on the cell body that the gap junctions most likely are formed (45).

In addition to the typical surface gap junction plaques, cytoplasmic gap junction vesicles have also been reported in the adrenal gland and in adrenal cell cultures with both transmission electron microscopic and immunocytochemical techniques (46–49). It has been confirmed with live cell imaging of cells expressing green fluorescent protein construct (Cx43-GFP) that these annular gap junction vesicles form from a unique process in which the gap junction membrane of one cell is internalized into the cytoplasm of the adjacent cell to form a double-membraned vesicle composed of gap junction protein. This internalization process occurs from the central regions of the gap junction plaques or, in some cases, the entire gap junction plaque membrane is internalized. Once internalized, the annular gap junction vesicle is degraded (50–52), and thus, this is a method for disassembling gap junction plaques and regulating communication. In contrast, gap junction plaque assembly occurs by the addition of new gap junction channels at the gap junction plaque periphery (53). Mechanisms for controlling cell–cell communication are thought to involve both the assembly of gap junction plaques at the cell surface and the disassembly of these plaques by an internalization process that results in annular gap junction vesicle release into the cytoplasm. The capacity to specifically analyze connexin protein distribution within specific compartments is particularly critical for the study of tissues, such as the adrenal, which have cells that express different steroidogenic enzymes and respond differently to stimuli, based on their specific zonal locations.

## Gap Junction Distribution in the Adrenal Cortex

In the human, the adrenal cortex can be divided into three morphologically and functionally distinct zones: the outer most zone, zona glomerulosa, and the inner zones, zona fasciculata (ZF) and zona reticularis (ZR). These zones are composed of cells that express different steroidogenic enzymes and thus produce different steroid hormones. Specifically, the ZF produces glucocorticoids, and the ZR produces androgens. Both of these inner zones secrete hormones in response to ACTH. The outer zone, however, produces aldosterone in response to changes in sodium, potassium, and the peptide hormone, angiotensin II.

**Figure 3:**
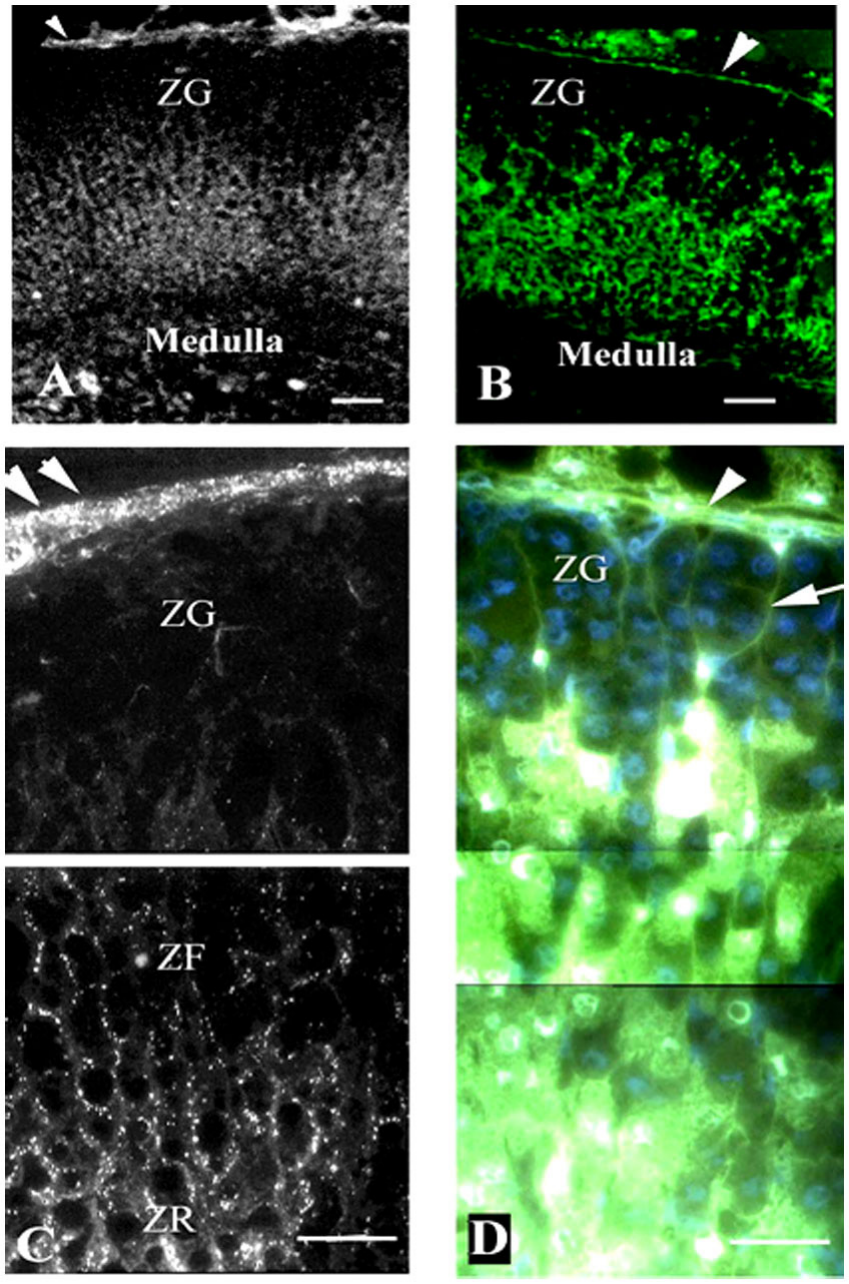
**Gap junction (Cx43) localization and dye communication in the intact adrenal gland**. Immunohistochemical localization of Cx43 gap junction proteins revealed extensive staining in the zona fasciculata (ZF) and zona reticularis (ZR), while there was limited staining in the zona glomerulosa (ZF) [left panels: **(A,C)**]. Correspondingly, lucifer yellow dye communication between cells was more abundant in the inner zones of the adrenal cortex (ACTH responsive areas) than in the outer zone [right panels: **(B,D)**]. Capsule (arrowheads), connective tissue trabecule (arrow), and Cx43 (white puncta). Bars: **(A,B)** 50 μm and **(C,D)** 30 μm [modified from Ref. (30)].

**Figure 4:**
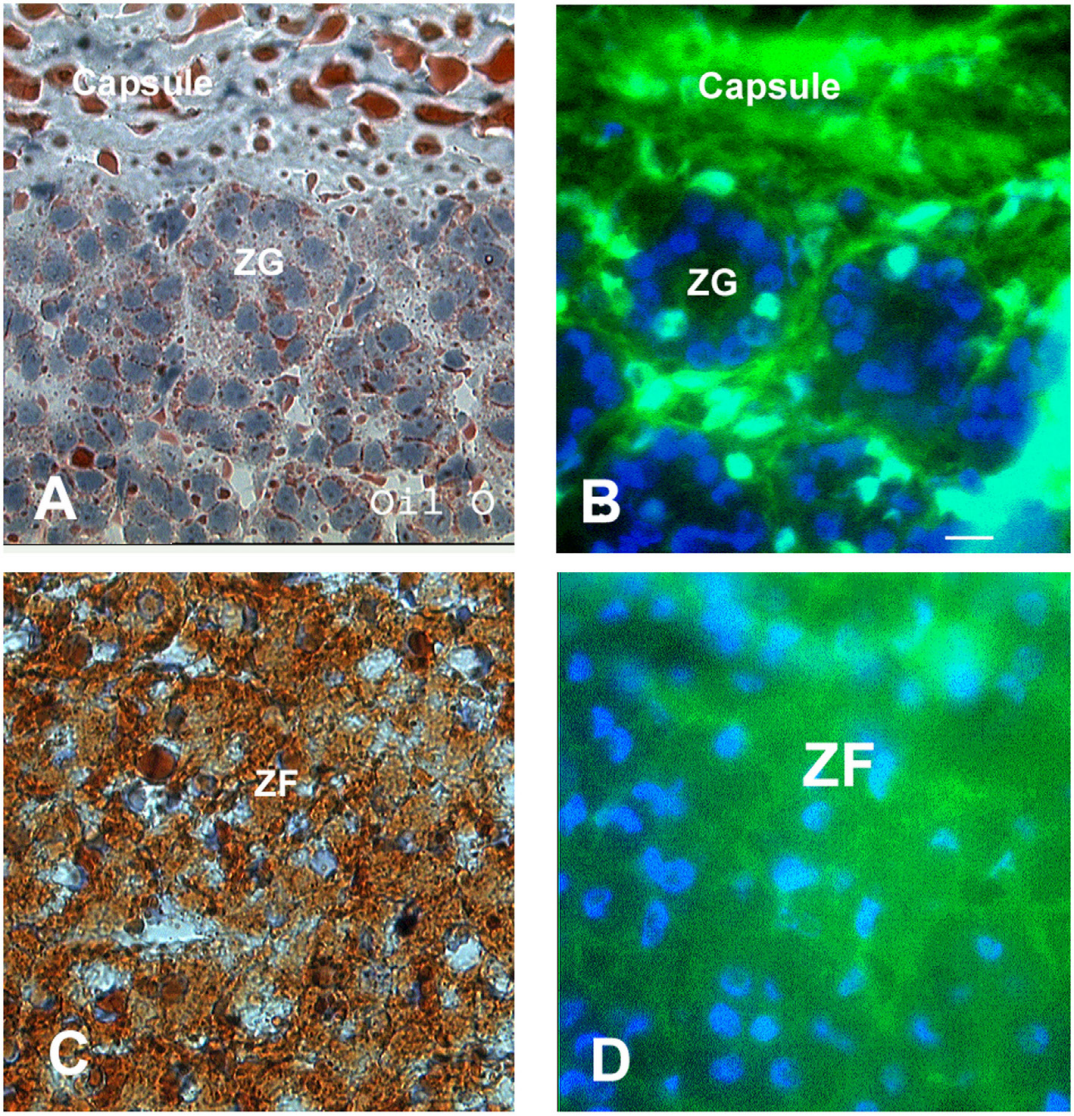
**Communication in the intact adrenal cortex**. Oil Red O staining was used to distinguish the lipid-rich zona fasciculata and zona reticularis from the zona glomerulosa **(A,C)**. Lucifer yellow dye was transferred between the fibroblasts of the connective tissue capsule and trabeculae; however, communication was absent between cells of the zona glomerulosa **(B)**. The cells of the inner two cortical zones, particularly those in the zona fasciculata, exhibited extensive dye communication **(D)**. Note the Lucifer yellow and Oil Red O staining are diffuse and seen throughout the cytoplasm, which somewhat obscures the cell boundaries and nuclei. Bars: **(A–D)** 30 μm [modified from Ref. (30)].

Just as the three zones of the adrenal have been demonstrated to be morphologically and functionally distinct, the level of expression of gap junction protein in the three zones of the adrenal has been demonstrated to differ using immunocytochemistry (Figures 3 and 4). Specifically, a differential distribution of Cx43 has been reported in the adrenal of human (4, 42) as well as a host of other mammals including rat (3, 5), mouse (40, 54), guinea pig (55), rhesus monkey (40), and cow (4, 8). Specifically, little or no Cx43 gap junction protein was detected between adrenal cells in the zona glomerulosa. In contrast, numerous gap junction plaques were evident at areas of cell–cell contact in the inner cortical areas, such as the ZF and ZR (Figure 3). The distribution of gap junctions once speculated from transmission electron and freeze-fracture microscopy (29, 55) could be confirmed and, more importantly quantitated, while simultaneously validating which zone was being viewed. With computer-assisted microspectrofluorometric image analysis, it was demonstrated that there were twice as many gap junctions per area between cells in the ZR than between cells in the ZF (6). While the number of gap junctions differed, there were no significant differences in the average size of gap junctions in the rat ZF compared with those in the ZR.

Consistent with the immunocytochemical quantitative analysis, when the expression of Cx43-encoded mRNA was analyzed with Northern Blot techniques in samples prepared from the capsule of the adrenal gland, which contain adherent zona glomerulosa cells, there was little gap junction RNA detected. In contrast, samples prepared from decapsulated glands, which contained mainly the ZF/ZR cells, had an abundance of gap junction RNA. It is thought that the detection of small amounts of Cx43-encoded mRNA in the zona glomerulosa with Northern Blot and almost no Cx43 gap junction proteins detected with immunocytochemical analysis may reflect possible contamination of the zona glomerulosa-enriched samples with cells from the ZF. In addition, some of the mRNA Cx43 detected with Northern Blot in the zona glomerulosa-enriched samples is thought to come from the presence of fibroblastic cells from the connective tissue capsule and trabeculea that project into the substance of the cortex in the sample (Figure 3D). In fact, these trabeculea within the substance of the gland were revealed with immunocytochemistry. To increase the accuracy for comparing the distribution of gap junctions with adrenal cortical zones, Oil Red O staining, which detects lipid droplets in the cytoplasm of cells in the ZF and ZR (56), has been used. The Oil Red O stain is much less abundant in zona glomerulosa cells that have few, if any, lipid droplets (56). The pattern of Oil Red O staining distinguished the cells of the zona glomerulosa from cells of ZF and ZR and substantiated that the cells which lack Cx43 gap junction staining are indeed cells of the zona glomerulosa (Figure 4) (30).

In parallel to the differential localization of gap junction plaques is the pattern of dye communication in the intact adrenal cortex. Specifically, lucifer yellow dye moved extensively in the inner cortex while dye communication was limited to the capsular projections of connective tissue between the cells of the zona glomerulosa (Figures 3B,D and 4B,D) (5, 30). Dye communication was not observed between cells of the zona glomerulosa. This lack of dye movement would be consistent with the observed absence of Cx43 protein in immunofluorescence studies and would further support the suggestion that other connexin family members do not assemble into functional gap junctions in this zone. In the inner two cortical zones, where gap junctions were plentiful, dye was transferred (30). It should be noted that while Cx43 was found in the inner two zones, it is possible that connexin types, other than Cx43, may be located in these inner two zones since not all of the 21 known connexin family members have been evaluated. In addition, there is a possibility that other, yet to be discovered, connexin type could be detected in the future. It is speculated that while cells of the zona glomerulosa, for reasons yet to be determined, are less dependent on cell–cell communication, the communication of a regulatory molecule between cells of the inner two layers modulates their functions.

## Role of Gap Junctions in Adrenal Cortical Function

### Steroidogenesis

Gap junctions, for years, have been suggested to play a key role in a number of physiological phenomena (3, 57, 58). However, in early years, this proposed role was solely based on the observations that dye and current could move between cells that had gap junctions and not on experimental evidence that demonstrates actual physiological responses. Adrenal cortical cells were one of the first endocrine cell types in which a direct relationship was established between gap junctions and physiological responses (59). Furthermore, the findings in the adrenal cortex have been used to lend support to the speculations that gap junctions influence physiological events in other cell types (60, 61).

**Figure 5:**
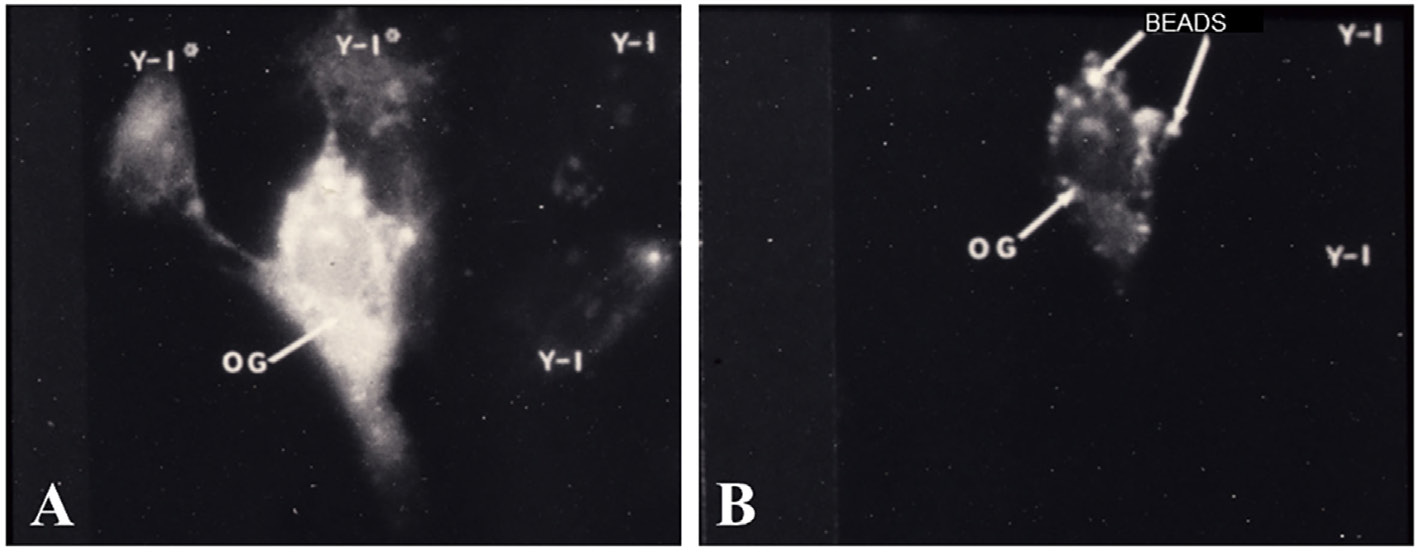
**Cocultured adrenal/granulosa cell pairs**. Adrenal cell clusters viewed with optics specific for fluorescein isothiocyanate **(A)** or rhodamine isothiocyanate **(B)** were treated with follicle-stimulating hormone (FSH) for 30 min. Abundant protein kinase dissociation was initially observed in granulosa cells (OG) and could be seen, after 5–15 min, in two adrenal cells (Y-1*) that are in contact with the granulosa cell, while two nearby adrenal cells (Y-1) that are not in contact with the granulosa cell failed to dissociate PKA **(A)**. Granulosa cells were identified by pre-labeling them with rhodamine-coated beads **(B)** [modified from Ref. (59)].

The first set of experiments, which demonstrated functional gap junction-mediated communication, were studies in which adrenal cells were placed into culture with ovarian granulosa cells. It is well documented that cells of the inner adrenal cortical zones possess specific receptors for ACTH and, when bound to its receptor, ACTH elicits a number of responses (62, 63), including an activation of cAMP-dependent protein kinase A (PKA) (59, 64), an increase in steroid synthesis (7, 65, 66), and a decrease in proliferation of cells in culture (2, 8, 67). In the adrenal/granulosa coculture populations, a cytochemical method was used to specifically localize free catalytic subunits (C) of PKA at subcellular, light microscopic levels of resolution (59, 68). In these studies, heterotypic, adrenal/granulosa, cell pairs were demonstrated to form gap junctions and ACTH treatment activated PKA in the adrenal cell populations (59). Moreover, ACTH stimulation not only, as expected, activated PKA in the adrenal cells but also with time, within the granulosa cells that were in contact with adrenal cells. The ovarian granulosa cells lacked ACTH receptors but had receptors for follicle-stimulating hormone (FSH), which activates PKA (69). Treatment of the coculture cell population with FSH activated PKA in the granulosa cells and in the adrenal cells that formed cell contacts with granulosa cells (Figure 5). Importantly, adrenal cell secretion of steroid hormones was increased (59). The finding that hormone treatment stimulated bidirectional exchange of a signal that initiated PKA activation in adrenal/granulosa coculture cell populations provided compelling evidence that the hormone-induced intercellular communication, which activated PKA, could serve a biological role.

In further support of the biological role of gap junctions in adrenal cortical function, inhibition of gap-junctional communication with 18-alpha glycyrrhetinic acid (18-alpha GA) treatment decreased the steroidogenic responsiveness of bovine adrenal cells in culture to ACTH (4). Specifically, it was demonstrated that 18-alpha GA treatment decreased the steroidogenic response to a submaximal dose of ACTH but did not decrease steroidogenesis if cells were treated with saturating doses of ACTH (4). A similar decrease in ACTH-stimulated steroidogenesis was demonstrated in bovine and human cell lines and in rat primary cell populations treated with 18-alpha GA (8). The effects of treatment with this glycyrrhetinic acid derivative were also substantiated in experiments in which Cx43 antisense transfection techniques were used to inhibit gap junction-mediated communication (8). Cell populations expressing Cx43 antisense grew faster, had decreased capacity for cell–cell communication, and had a diminished steroidogenic responsiveness to ACTH treatment compared with null transfected control populations (8). This decrease in ACTH-mediated steroidogenesis following gap junction inhibition by either chemical treatment or Cx43 antisense expression is consistent with the concept that gap junction-mediated communication is a necessary factor in hormonal response. It has been suggested that gap junction communication of cAMP signaling, and subsequent PKA activation in the recipient cell, may increase the efficiency of hormone response by facilitating amplification of ACTH signaling throughout the population. This would be particularly useful at submaximal stimulation doses of the hormone, as suggested by Munari-Silem, when only a small population of the cells may be responding to ACTH (4).

In addition to 18-alpha, 18-beta glycerrhetinic acid (18-beta GA) has also been used in a number of studies to inhibit gap junction communication (70–72). Huang and colleagues found that treatment with 18-beta GA increased the basal levels of steroidogenesis secreted in rat primary adrenal cell cultures (73). These findings are interesting given that the increased steroidogenesis appears to be PKA-independent and would suggest a direct effect of the 18-beta GA on the production of steroidogenic enzymes in addition to their effect on inhibition of gap junction-mediated cell communication. It should be noted that neither cell communication nor the effect of ACTH stimulation was measured in this study. Further investigations are needed to determine if the concentrations of 18-beta GA used in this study eliminated cell–cell communication, and more importantly, if the ACTH-stimulated steroidogenesis at submaximal and saturating treatment doses were altered. It should also be noted that both 18-alpha and 18-beta GA have formulas very similar to that of cortisol and may be mistaken, with some assay methods, for an adrenal steroid (74).

### Proliferation

In the intact adrenal gland, it was noted that the cells of the zona glomerulosa, which had few if any gap junctions, divided rapidly. In contrast, cells of the inner zones, where gap junctions were more abundant, proliferated more slowly (6, 40). Based on this inverse relationship between the rate of proliferation in the adrenal zones and the presence of gap junctions, it was suggested that gap junctions play a role in regulating cell proliferation in the adrenal cortex.

In the zona glomerulosa, it has been suggested that the lack of gap junctions and thus capacity for direct communication of growth regulatory molecules would result in more cell proliferation in this zone. Along these same lines of evidence, the slower proliferation rate of cells in the inner zones, where gap junctions are more abundant (5, 40), would be consistent with their greater capacity to communicate growth inhibitory signals. Many factors are known to regulate growth, and gap junction-mediated communication may play a key role by facilitating the movement of these factors between the cells.

In support of the hypothesis that gap junctions contribute to the control of adrenal cell proliferation, possibly by allowing passage of molecules between cells, are the findings that cell proliferation of bovine adrenal cortical cell (SBAC) populations was significantly increased if gap junction protein expression was suppressed by Cx43 gap junction cDNA antisense transfection techniques (8). The average number and size of gap junction plaques decreased while the rate of population growth increased in these studies (8). Conversely, when adrenal cells were treated with ACTH, which increased gap junction protein expression, there was a corresponding decrease in cell population growth (8). Both the ACTH-stimulated increase in gap junction protein expression and decrease in cell population growth were mimicked by treatment with the second messenger, dibutyryl cyclic adenosine monophosphate [DbcAMP (1 mM)]. It is, thus, thought that the ACTH-induced alterations in Cx43 gap junction protein expression and proliferation are modulated by a PKA mechanism. Further, the findings of an inverse relationship between gap junctions and proliferation would suggest that, in the adrenal cortex, along with the multiple mechanisms known to be involved in controlling adrenal gland structure and function, ACTH-stimulated intercellular communication *via* gap junctions may represent an important factor in adrenal gland behavior. However, not only is gap junction-mediated cell communication thought to regulate ACTH-induced changes in steroidogenesis and proliferation but also there is compelling evidence that ACTH serves to regulate gap junction protein expression and stability at the cell surface.

## Regulation of Gap Junction Expression

In a number of studies of adrenal cells in culture, ACTH has been demonstrated to increase gap junction protein expression and to increase the size and number of surface gap junction plaques (4, 7, 8). These effects on gap junctions can be mimicked by treatments that increase cAMP levels and activate PKA (59, 64). Specifically, an increase in gap junction plaque size and number at the cell surface as well as the decrease in gap junction plaque disassembly (internalization to form annular gap junctions) was reported following DbcAMP treatment. These results are consistent with the theory that the changes in gap junctions following ACTH treatment were dependent on cAMP and the activation of protein kinase. Conversely, elevation of cAMP and PKA activation has been demonstrated to decrease the number of annular gap junctions in adrenal cortical cells (75). This suggests that PKA activation may decrease gap junction plaque internalization, which would further contribute to the observed increase in gap junction plaques at the cell surface following treatments that elevate cAMP levels in adrenal cell populations.

**Figure 6:**
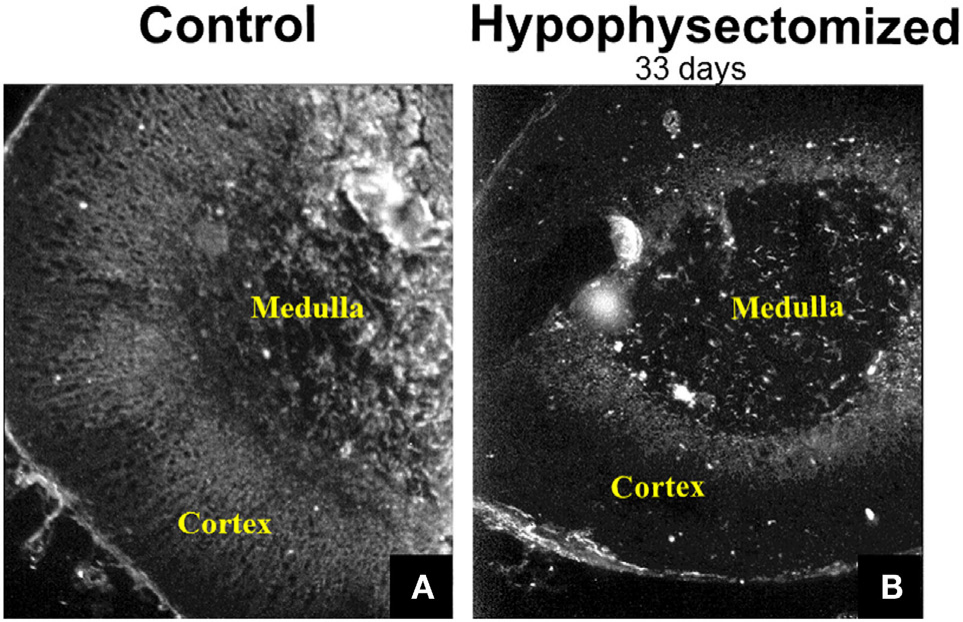
**Immunohistochemical demonstration of gap junction (Cx43) distribution in the adrenal gland**. Gap junction proteins were abundant in the inner cortex of the mouse adrenal **(A)**. Hypophysectomy led to diminished Cx43 gap junction expression mainly in the zona fasciculata at 33 days post-surgery **(B)** [modified from Ref. (31)].

In addition to the findings made in adrenal cell cultures, the relationship between gap junction protein expression, occurrence, distribution, and ACTH levels in the body have been evaluated in studies in which the tropic state of the adrenal gland was altered by surgical removal of the pituitary (46). The removal of the pituitary, termed hypophysectomy, eliminates the source of ACTH since the pituitary secretes this as well as a variety of other hormones that are either produced by cells of the anterior pituitary (growth hormone, gonadotrophins, prolactin, and thyroid stimulating hormone) or by cells of the hypothalamus and then stored in the pituitary (antidiuretic hormone and oxytocin) (1, 76). The elimination of ACTH by hypophysectomy led to a profound atrophy of the cortex, which was more marked in the inner zones (ZF and ZR) than in the zona glomerulosa (46). While increasing ACTH levels increased gap junctions, eliminating ACTH by perturbing the pituitary–adrenal gland axis by hypophysectomy in mice led to diminished Cx43 gap junction expression mainly in the ZF (Figure 6) (31). If these hypophysectomized animals were treated with ACTH, Cx43 gap junction plaque size was increased (46). The increase in gap junction protein expression occurred in the ACTH-dependent zones (ZF and ZR) with no change in the ACTH-independent zone (zona glomerulosa). Thus Cx43 gap junction protein expression can be regulated *in vivo* as well as in adrenal cell populations maintained in culture, in further support that ACTH can modulate gap junctions.

The molecular mechanism for how ACTH may modulate gap junctions has not been demonstrated in the adrenal gland. It is well documented, however, that ACTH treatment results in PKA activation (64); reviewed by Ruggiero and Lalli (77), and in a number of other cell types, activation of PKA has been shown to increase gap junction plaque assembly by facilitating the phosphorylation of gap junction protein (78). Specifically, PKA activation results in the phosphorylation of serines (S364, S365, S369, and S373) on the C-terminal tail of Cx43 (78). Treatment with ACTH, based on this information, would be expected to phosphorylate one or more of these serines. There is a need, however, for increased information on the relationship between ACTH treatment and connexin phosphorylation if we are to fully understand the role of gap junction function in adrenal cell behavior.

To date, the largest increase in information on gap junction regulation and function comes from the knowledge of which Cx43 C-terminal tail amino acids are phosphorylated and dephosphorylated (22, 78–81). Phosphorylation/dephosphorylation events on the C-terminal tail of Cx43 are dependent on activation of PKA, as well as a number of other kinases including protein kinase C (PKC), tyrosine kinase, mitogen-activated protein kinase (MAPK), and casein kinase 1 (22, 82–84). These protein kinases, through triggering the phosphorylation of particular amino acids on the tail of Cx43, regulate gap junctions (assembly into plaques, stability at the cell surface, gap junction plaque disassembly, and cell–cell communication). Specifically, there are at least 11 serine and 2 tyrosine residues on the C-terminal tail of Cx43 (Cx43-C-terminus) that, when phosphorylated, result in either an increase in gap junction function (S325, S328, S330, S364/365, and S373) or the downregulation of gap junction activity (Y247, S255, S262, Y265, S279/282, and S368) (85–87). These observations have been made mainly from studies in clonal lines of murine fibroblasts (L929 cells), fibroblasts derived from Cx43 knockout and wild-type mice, rat liver epithelial cells (T51B), HeLa cells, and rat primary granulosa cells (88). It should be noted that ACTH-mediated activation of either S364, S365, S369, S373, or other possible amino acids of the C-terminal tail of Cx43 has not, to our knowledge, been examined. Furthermore, protein kinase-mediated phosphorylation of the C-terminus of Cx43 has been demonstrated to play a role in gap junction plaque internalization and annular gap junction vesicle formation (89, 90). Specifically, MAPK, casein kinase 1, and PKC activation increases the number of annular gap junction vesicles while decreasing gap junctions on the cell surface (69, 80, 89–92). In the case of PKC-mediated hyperphosphorylation of Cx43, it is thought that phosphorylation makes this connexin more vulnerable to proteolytic degradation, thus decreasing gap junction-mediated communication. The interplay between the various kinase pathways in Cx43 phosphorylation and cell–cell communication have not been elucidated in the adrenal cortex. Such studies, however, are needed if we are to fully understand the role of ACTH in regulating those endocrine cell responses that are dependent on the communication of molecules between cells. Certainly, in the adrenal gland, there is a need for cells to cooperate and get information from one another. Gap junctions provide this function, and when gap junctions are lost from a population, pathological conditions, including cancers, are thought to develop (42, 93, 94).

## Loss of Gap Junctions and Adrenal Cortex Cancer

The loss of gap junction function has been implicated in tumor development (93–99). Most tumors associated with the adrenal cortex develop in the ZF (100) where, under normal conditions, gap junctions tend to be large and abundant (6, 40, 42). The most common types of adrenal gland tumors are adrenal adenomas, which are non-cancerous tumors of the cortex (101, 102). Adrenocortical carcinoma, cancer of the adrenal cortex, is rarely observed; however, patients with certain inherited genetic disorders are at a higher risk of developing this cancer (102).

In the adrenal cortex, a decrease in gap junction expression has been correlated with the stage of tumor differentiation/progression (42). Specifically, in studies to characterize gap junctions during tumorigenesis, the number of gap junctions observed per cell was quantitated and compared in normal human cells of the adrenal gland ZF, as well as cells from adenoma and carcinoma adrenal gland tissues. Cells from the ZF of normal adrenal glands displayed a much higher number of gap junctions per cell (13.78 ± 1.93 SEM) than when compared with benign adrenocortical adenomas (4.6 ± 1.17 SEM; *p* ≤ 0.05). The number of gap junctions demonstrated between cells of malignant adrenocortical tumors (1.42 ± 0.58 SEM; *p* ≤ 0.05) were significantly lower than both the normal and benign cells (42). These observations are consistent with the hypothesis that a capacity for increase in proliferation coupled with the loss of terminal differentiation as the tumor progresses from non-malignant to malignant reflects the loss of gap junction-mediated communication of growth regulatory molecules between cells. Further, it has been suggested that the increased capacity for tumor metastasis may result from the loss of the cell–cell adhesion that is provided not only by adhesion junctions but also by gap junctions (103–105). Interestingly, exogenous Cx43 can decrease cell proliferation and contribute to reversion of the transformed phenotype (106, 107). Both observations are suggestive of a relationship between gap junctions, differentiation, and proliferation. There is a hope that the induction of gap junctions in malignant cells will provide a novel therapeutic strategy for treating adrenal cancer and, in addition, pharmacological methods and manipulations designed to increase gap junctions may someday serve as a therapeutic method.

While some investigators have suggested the loss of gap junctions in adrenal tumor cell populations, others have demonstrated that metastatic tumors are correlated with an increase in the expression of connexins dependent on the tissue type (108–111). In few cases, tumor cells are capable of communication with other tumor cells through gap junctions; however, they do not communicate with normal cells (112). The difference in the detection of changes in some studies compared with others may reflect the complex nature of cancers in addition to the adrenal zones of origin of the various tumors. Further investigations of the relationship between gap junction expression and tumor progression are needed. This is specifically true when studying the adrenal gland, given the number of adrenal incidentalomas that are commonly detected and the need to differentiate benign from malignant detections. The reported lack of gap junctions in the carcinoma cell population, if found to be widespread in different tumor populations, could be used as an additional method of determining the capacity for metastasis and as an indicator of the rate at which the tumor may proliferate. Furthermore, if cancer cells of the adrenal are capable of communicating with one another but not with normal cells as suggested by Yamasaki in other tissues (112), it would be possible to design protocols that use gap junction-mediated communication to selectively kill the cancer cells while leaving the normal healthy cells intact, the bystander effect (113–115).

## Cross Talk Between Cells of the Cortex and Medulla

It is becoming clear that the cells of the medulla and the cortical cells are physically interwoven with one another (116) and, thus, the possibility of gap junction-mediated communication between chromaffin cells and adrenocortical cells may exist. In humans, as well as other mammals, for example cortical cells can be found within the medulla in contact with chromaffin cells (116–118). Furthermore, inner cortical cells of the ZR are in direct contact with the chromaffin cells of the medulla (1), and gap junctions between these cells could possibly provide a mechanism for direct communication of information. Certainly, there is a functional relationship between the cortical and medullary cells, since the adrenal androgens, particularly dehydroepiandrosterone (DHEA), which is produced in ZR following ACTH stimulation, can inhibit chromaffin cell proliferation and differentiation (119, 120). In addition, cells of the cortex may influence catecholamine release from chromaffin cells while the chromaffin cells may regulate adrenal cortical steroid hormone production (10, 116, 121). Gap junctions may provide a mechanism for some of the functional interdependency, especially, among those cells at the medullary–cortical interface area and among islands of cortical cells located within the medulla.

In the medulla, several different connexin types (Cx29, Cx36, Cx43, and Cx50) have been reported to be expressed, depending on the species (41, 122). While some investigators have not reported gap junction plaques in the medulla, others have demonstrated clusters of medullary cells that express Cx43 antigens (5). It is thought that gap junctions in the medulla play a role in the catecholamine (epinephrine and norepinephrine) secretory process and, thus, helps in mediating the body’s response to stress (54, 123–125). In addition, stress causes a well-orchestrated, cascade of events that result in the release of a number of other hormones, including glucocorticoids from the adrenal cortex (126). Gap-junctional coupling may complement electrical coupling to enhance communication of signals needed for synchronized cellular hormone release in response to ACTH or low synaptic activity during stress (125).

## Summary

**Figure 7:**
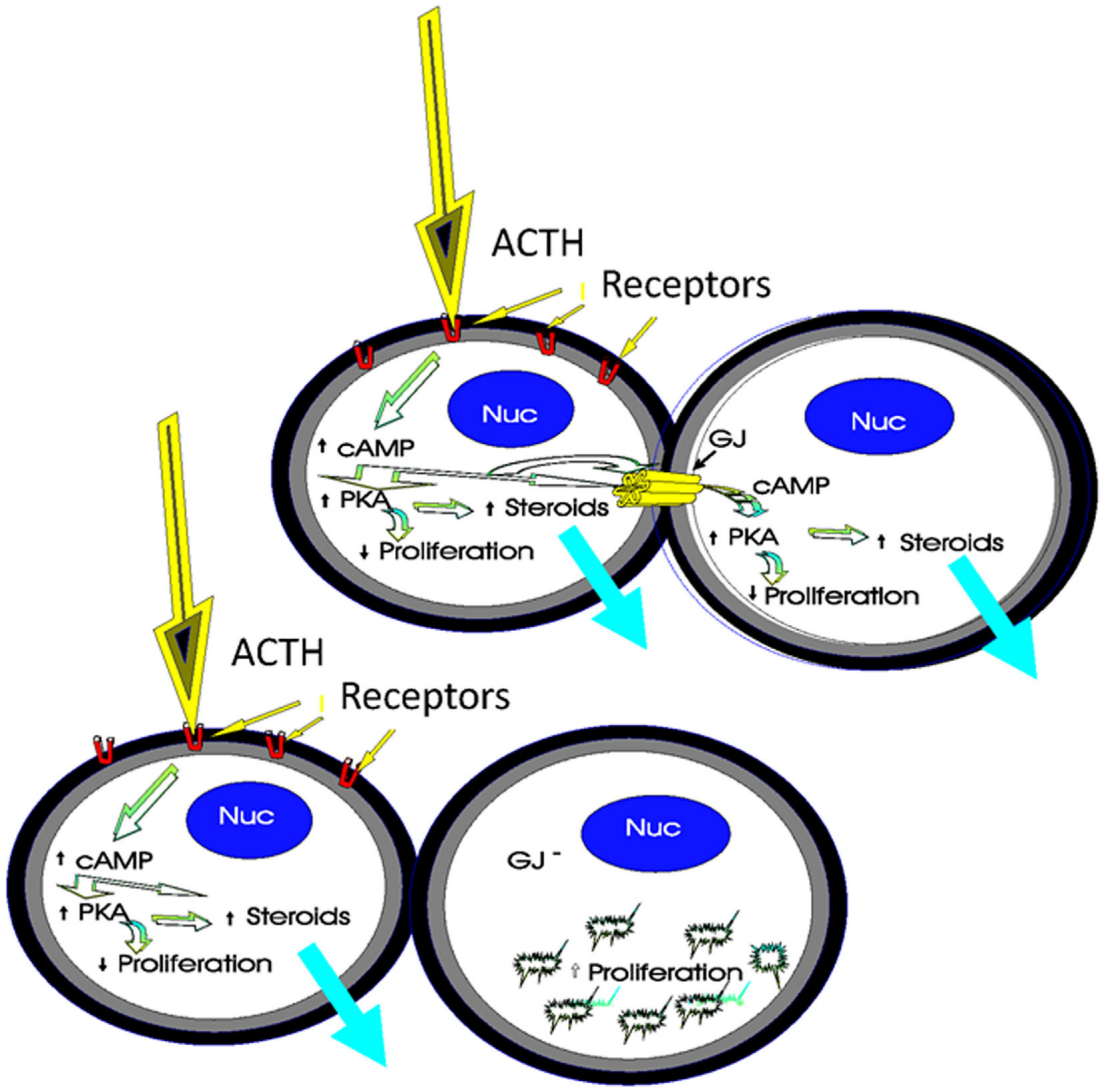
**Schematic of the role of gap junctions in steroidogenesis and cell proliferation**. The binding of ACTH to its receptors on coupled cortical cells stimulates cyclic adenosine monophosphate (cAMP) to activate protein kinase A (PKA), thereby increasing steroidogenesis and decreasing proliferation. Gap junction-mediated movement of cAMP between cells in the population would amplify hormonal responses in contacting cells, thus increasing ACTH-mediated steroidogenesis. An increase in the number of gap junctions, increases ACTH-mediated steroidogenesis while decreasing proliferation. However, if gap junctions are decreased as a result of molecular manipulations or chemical treatments, for example, ACTH stimulation in one cell does not affect steroidogenesis in the second cell, and there would be a loss in coordinated cell function between cells [modified from Ref. (130)].

Gap junctions are thought to be important in regulating cell population growth, cell morphology, differentiation, cell migration, wound healing, and cell function (2, 93, 108). By analyzing the frequency, distribution, and function of these junctions within intact adrenal glands as well as in adrenal cells in culture following ACTH stimulation, the role of connexins in these processes may be clarified. The specific spatial abundance of gap junction proteins in the adrenal cortical zones is intriguing with regard to its functional implications. It can be suggested that cells of the zona glomerulosa, which express fewer gap junctions than the two other cortical zones, may be less dependent on cell–cell communication for normal function than the cells of the ZF or ZR. Further, a relationship between proliferation rates, steroidogenesis, and gap junction presence or absence in the gland has been suggested (Figure 7). Studies to demonstrate the effects of gap junction overexpression and inhibition in the adrenal glands are needed, if we are to resolve some of the questions of how gap junctions function as adrenal cells undergo the morphological and proliferative changes associated with the establishment and maintenance of cortical zonation and hormone-mediated steroidogenesis.

Intact adrenal glands and adrenal cell cultures provide us with a valuable paradigm for studying gap junctions and the cellular mechanisms involved in hormone responses. Indeed, study of adrenocortical cells *in vivo* and *in vitro* is important not only for an understanding of adrenal gland function *per se* but also for providing insight into the foundation of cell communication and biological response in other tissues. But, beyond cell communication, it is becoming apparent that gap junctions play a role in providing adhesive forces needed to hold cells together (103–105).

In addition to gap junction-mediated communication and adhesion, the gap junction internalization process that results in increased annular gap junction vesicles within the cytoplasm and fewer surface gap junction plaques could be important to events in the adrenal gland. Annular gap junctions were reported in the adrenal gland as early as 1988 with transmission electron microscopy (49). It has been demonstrated that annular gap junctions undergo degradation (13, 51, 52). Relatively large numbers of annular gap junctions occur in the adrenal gland, and it could be postulated that gap junction turnover is high in this tissue. Rapid turnover could be needed for migration of the cells to maintain the cortical zones during cytogenesis, for example. Such suggestions are not without precedent. In the ovary, for example, gap junctions are thought to allow the movement of molecules to the egg from the follicular granulosa cells (60, 61). In the case of the ovary, phosphorylation of the C-terminal tail of connexin has been demonstrated to be regulated by the peptide hormone, luteinizing hormone (127). Large numbers of annular gap junctions are reported during ovulation, and it is thought that if gap junctions are not internalized from the surface of the granulosa cells that the egg will remain “trapped” within follicle and ovulation will not occur (128). Gap junctions in a similar way could potentially retard the migration of cells from one area to another (centripetal migration) by tethering the cells to one another. Inhibiting cell migration through the granulosa cell layer of the follicle, in the case of the ovary, or as cells migrate in the adrenal gland could keep cells connected to one another if gap junctions fail to internalize. Such possibilities, however, are yet to be demonstrated.

## Remaining Questions

It is clear from early studies that there is a relationship between gap junctions and ACTH-mediated responses in the adrenal cortex. There are, however, very few recent articles available on gap junctions in the adrenal cortex. Yet, there are important questions that remain to be answered. For example, in human conditions with diminished circulating ACTH levels, are there changes in the expression or distribution of adrenal cortical gap junctions, as seen following hypophysectomy in rodents (30)? There is dramatic remodeling and relocation of gap junction plaques in the diabetic heart (129). Would the remodeling of gap junctions in the adrenal occur in this and in other endocrine related diseases? Adrenal cortical steroidogenesis is influenced mainly by the activation of PKA (59, 64). In tissues, other than the adrenal, PKA has been shown to phosphorylate the C-terminal tail of Cx43 gap junction proteins (22, 78–81). Such PKA-mediated connexin phosphorylations have been demonstrated to be critical in regulating gap junction assembly and stability (22, 78–81). In the adrenal cortex following ACTH treatment, it is presumed that gap junction protein phosphorylation occurs. However, which Cx43 C-terminal tail amino acids are phosphorylated is unknown. Further, the interplay between PKA and the other protein kinases (PKC, tyrosine kinase, MAPK, and casein kinase 1) in the regulation of gap junctions and adrenal cortical functions needs to be investigated. It has become clear that connexins can have multiple regulatory functions, which depend upon their phosphorylated state (22, 82, 84). Unfortunately, the role of protein kinase-mediated connexin phosphorylation in the adrenal cortex, although described in other tissues, has not been investigated, to our knowledge, in the adrenal cortex. However, one would predict that as cells alter their function that the need for gap junction-mediated cell communication, and thus gap junction expression and phosphorylation, may also change. Moreover, the molecules moving between cells may change. One molecule that has been demonstrated to transfer between gap junctions of adrenal cells to regulate steroidogenesis is cAMP (59, 68). But surely, more than this, one molecule is moving between the cells of the adrenal. The abundance of gap junctions in the inner cortex would suggest a high demand for communication of molecules in these zones. The following question has yet to be answered: how movement of molecules between cells serves to regulate the cascade of events involved in proliferation but more importantly in differentiation of the cells such that transcription factors needed for the production of cortisol in one zone of the adrenal but for androgens in another zone.

The study of adrenocortical cells *in vivo* and *in vitro* is important not only for an understanding of adrenal gland function *per se* but also for providing insight into the foundation of cell communication and biological response in other tissues. Further study will elucidate how gap junctions and cell–cell communication as well as gap junction-mediated adhesion is related to adrenal gland development cell differentiation and steroidogenic function.

## Author Contributions

CB wrote sections of the manuscript, prepared figures, and edited the manuscript. SM wrote sections of the manuscript, prepared figures, and edited the manuscript.

## Conflict of Interest Statement

The authors declare that the research was conducted in the absence of any commercial or financial relationships that could be construed as a potential conflict of interest.

## Abbreviations

18-alpha/beta GA, 18-alpha/beta glycerrhetinic acid; ACTH, adrenocorticotropin hormone; DbcAMP, dibutyryl cyclic adenosine monophosphate; FSH, follicle-stimulating hormone; GFP, green fluorescent protein; LAMP, lysosomal-associated membrane protein; MAPK, mitogen-activated protein kinase; PKA, protein kinase A; PKC, protein kinase C; RNAi, RNA interference; SBAC, bovine adrenal cortical cell line; SW-13, Scott white human adrenal cortical tumor cell line; TEM, transmission electron microscopy; TPA, 12-*O*-tetradecanoylphorbol-13-acetate; Y-1, mouse adrenal cortical tumor cell line.
